# Primary Ovarian Pregnancy

**Published:** 1901-01-19

**Authors:** 


### PRIMARY OVARIAN PREGNANCY.

At the last meeting of the Obstetrical Society Mr. G. P. Anning and Mr. Harry Littlewood, of Leeds, communicated a paper in which they described a case of primary ovarian pregnancy with rupture fourteen days after the last menstruation. The case was one of much interest from the fact that, although it had long been believed that ovarian pregnancy was possible, a more careful investigation of the reported cases had of late years led some to the opposite conclusion. This well recorded case, therefore, marks a point in the history of ectopic gestation, giving proof, as it does, of the possibility of ovarian pregnancy. The patient was 28 years of age and had been married five months. There had been no previous pregnancy. Menstruation was normal. On August 27th she was operated upon for a ruptured ectopic gestation about thirty-six hours after the rupture. About two pints of blood and clot were removed, and a small ovum about the size of a Barcelona nut was found. This fitted into a firm envelope composed of laminated clot. There was a rent in the right ovary leading to a cavity which contained some blood; into this cavity the ovum and its sac exactly fitted, indicating the primary ovarian origin of the pregnancy. The right tube was removed and showed no evidence of rupture. The left tube was examined and found normal. The patient made a good recovery. Microscopical sections were shown of the ovum, the sac, and a portion of the ovarian wall. In commenting on this case Mr. Bland Sutton insisted upon the weakness of the evidence which, before 1899, had been brought forward in proof of ovarian pregnancy, and said that, after a careful examination of a large number of specimens he had come to the same conclusion as had been arrived at by Dr. Farre, namely, that although he would not deny the possibility of a spermatozoön entering an ovarian follicle, he had denied that any case of ovarian gestation had been satisfactorily proved. His investigations had been particularly directed to the very early stages, for he had urged as a postulate that if the ovum were really capable of being fertilised in its follicle "an early embryo in its membranes contained in a sac in the ovary" ought to be forthcoming. He was convinced, however, that the case now before the Society was a case of early ovarian pregnancy. Mr. Anning and Mr. Littlewood were, he said, entitled to and would receive the thanks of the Society for their kindness and energy in coming from Leeds and recording a case which would become historic. Mr. Bland Sutton then mentioned that he had recently visited Amsterdam, and through the courtesy of Dr. Catharine van Tussenbroek, had been able to see the early example of ovarian pregnancy described by her. This specimen he described, mentioning in particular that the condition of the parts was exactly analogous to that of a tubal mole, so that for convenience they would have to speak of it as an "ovarian mole." Another matter, he said, was the fact that the "mole," with its chorionic villi, which had become the criterion for many cases of early tubal pregnancy, seemed likely from this specimen to become the criterion of early ovarian pregnancy also. A large field of inquiry had thus been opened, for the condition known as blood cysts of the ovary would now require very careful investigation in the new light afforded by this remarkable specimen. Dr. Galabin said that he had always believed in the possibility of both ovarian and primary abdominal pregnancy, and thought that those who denied it had gone upon the *a priori* assumption that the tube was the only structure besides the uterus which could give a primary attachment to the ovum.

